# Effect of hypoxia on the expression of αB-crystallin in head and neck squamous cell carcinoma

**DOI:** 10.1186/1471-2407-14-252

**Published:** 2014-04-11

**Authors:** Chantal van de Schootbrugge, Elisabeth MJ Schults, Johan Bussink, Paul N Span, Reidar Grénman, Ger JM Pruijn, Johannes HAM Kaanders, Wilbert C Boelens

**Affiliations:** 1 Department of Biomolecular Chemistry, Institute for Molecules and Materials and Radboud Institute for Molecular Life Sciences, Radboud University Nijmegen, 271, RIMLS, PO Box 9101, 6500 HB Nijmegen, The Netherlands; 2 Department of Radiation Oncology, Radboud University Nijmegen Medical Centre, PO Box 9101, 6500 HB Nijmegen, The Netherlands; 3 Department of Otorhinolaryngology–Head and Neck Surgery, Turku University Hospital, University of Turku, PO Box 52, FI-20521 Turku, Finland

**Keywords:** CRYAB protein, HspB5, Carcinoma, Squamous cell of head and neck, Hypoxia, Reactive oxygen species, Hypoxic cell survival

## Abstract

**Background:**

The presence of hypoxia in head and neck squamous cell carcinoma (HNSCC) is associated with therapeutic resistance and increased risk of metastasis formation. αB-crystallin (HspB5) is a small heat shock protein, which is also associated with metastasis formation in HNSCC. In this study, we investigated whether αB-crystallin protein expression is increased in hypoxic areas of HNSCC biopsies and analyzed whether hypoxia induces αB-crystallin expression *in vitro* and in this way may confer hypoxic cell survival.

**Methods:**

In 38 HNSCC biopsies, the overlap between immunohistochemically stained αB-crystallin and pimonidazole-adducts (hypoxiamarker) was determined. Moreover, expression levels of αB-crystallin were analyzed in HNSCC cell lines under hypoxia and reoxygenation conditions and after exposure to reactive oxygen species (ROS) and the ROS scavenger N-acetylcysteine (NAC). siRNA-mediated knockdown was used to determine the influence of αB-crystallin on cell survival under hypoxic conditions.

**Results:**

In all biopsies αB-crystallin was more abundantly present in hypoxic areas than in normoxic areas. Remarkably, hypoxia decreased αB-crystallin mRNA expression in the HNSCC cell lines. Only after reoxygenation, a condition that stimulates ROS formation, αB-crystallin expression was increased. αB-crystallin mRNA levels were also increased by extracellular ROS, and NAC abolished the reoxygenation-induced αB-crystallin upregulation. Moreover, it was found that decreased αB-crystallin levels reduced cell survival under hypoxic conditions.

**Conclusions:**

We provide the first evidence that hypoxia stimulates upregulation of αB-crystallin in HNSCC. This upregulation was not caused by the low oxygen pressure, but more likely by ROS formation. The higher expression of αB-crystallin may lead to prolonged survival of these cells under hypoxic conditions.

## Background

In solid tumors, hypoxic regions can be present when cells are exposed to an oxygen pressure below 5 to 10 mmHg (0.66 – 1.32% O_2_) [1]. Hypoxia can be a result of insufficient oxygen transportation to remote parts of a tumor, caused by deficient blood vessel formation (chronic, diffusion-limited hypoxia) or leaking or partially blockage of blood vessels (acute, perfusion-limited hypoxia) [1]. Hypoxia might be intermittent when the blood flow is restored after temporary vascular shutdown, which can result in a cycling pattern of hypoxia and reoxygenation [2-4]. The presence of hypoxic regions in the tumor is detrimental for the patient, since hypoxic tumor cells are associated with therapeutic resistance and metastatic progression [5-7]. Despite the low oxygen levels, hypoxia is also associated with the presence of reactive oxygen species (ROS) [8-10]. As ROS are conventionally thought to be cytotoxic and mutagenic, they could lead to cancer progression and might be one of the reasons why the presence of hypoxia is as a bad prognostic factor [11].

αB-crystallin is a small heat shock protein, which can bind to partially unfolded proteins, thereby keeping them in a soluble state to prevent their aggregation [12,13]. It may protect cells from death induced by accumulation of unfolded proteins [14]. Furthermore, αB-crystallin may confer stress resistance to cells by inhibiting the processing of the pro-apoptotic protein caspase-3 [15]. Besides being mainly expressed in eye lens and muscle tissues [16], αB-crystallin can also be found in several types of cancer, among which head and neck squamous cell carcinoma (HNSCC) [17-19] and breast carcinomas [20-22]. αB-crystallin expression is associated with metastasis formation in HNSCC and in breast carcinomas [19,23] and in other types of cancer, expression is often correlated with poor prognosis as well [12,13]. The expression of αB-crystallin can be increased during various stresses, like heat shock, osmotic stress or exposure to heavy metals [24]. Moreover, in tissues from newborn piglets, αB-crystallin has been shown to be upregulated by hypoxia [25,26]. In this study, we analyzed whether the expression of αB-crystallin protein is affected in hypoxic regions of HNSCC’s and whether αB-crystallin knockdown influences cell survival under hypoxic stress.

## Methods

### Patients

Biopsy material of 38 HNSCC patients with stage II to IV primary squamous cell carcinoma of the oral cavity, oropharynx, hypopharynx or larynx was used (not all biopsies of the available cohort could be used due to the lack of material) [19,27]. Two hours before biopsies were taken (1 per patient), patients received 500 mg/m^2^ body surface of the hypoxia marker pimonidazole (intravenously, dissolved in 100 ml 0.9% NaCl) over 20 minutes. The obtained biopsies were snap-frozen and stored in liquid nitrogen until immunohistochemical processing. Approval from the ethics committee of Radboud University Nijmegen Medical Centre was obtained and all patients provided written informed consent.

### Immunohistochemisty

Sections of the biopsies (5 μm) were mounted on poly-L-lysine coated slides, fixed for 10 minutes in acetone at 4°C and rehydrated in PBS. The sections were incubated overnight at 4°C with 100-fold diluted αB-crystallin antiserum [28] and subsequently incubated for 30 minutes at 37°C with 600-fold diluted FabCy3 goat-α-rabbit IgG (Jackson Immuno Research Laboratories Inc) in PBS for 45 minutes at 37°C with 10-fold diluted endothelium antibody PAL-E (Euro Diagnostica BV) in Primary Antibody Diluent (PAD, Dako) and for 60 minutes at 37°C with 100-fold diluted Alexa 647 chicken-α-mouse IgG (Molecular probes) in PBS. For visualization of the pimonidazole adducts, the sections were stained with 1000-fold diluted rabbit-α-pimonidazole (gift from Dr. James A. Raleigh, University of North Carolina), diluted in PAD for 30 minutes at 37°C and subsequently stained with 600-fold diluted Alexa 488 donkey-α-rabbit IgG (Molecular probes) in PBS 30 minutes at 37°C. During the latter step only the rabbit antibodies directed to pimonidazole were stained [29]. Between the incubations, 3 times 2 minutes washing steps in PBS were performed. The sections were mounted using fluorostab (ProGen Biotechnik GmbH).

### Image acquisition

Scanning of the biopsy sections was performed with a fluorescence microscope (Axioskop, Zeiss) and a computer-controlled motorized stepping stage, using IP-lab software (Scanalytics), as described previously [30]. Each section was completely sequentially scanned for αB-crystallin, pimonidazole and blood vessel staining at 100× magnification. The resulting composite grey scale images were converted to binary images for further analysis. Thresholds were set just above the background staining for each individual staining. The total tumor area was contoured manually, excluding nontumor tissue, large necrotic areas and artifacts. The percentage of αB-crystallin in the normoxic area was determined as the pimonidazole-negative tumor area containing αB-crystallin relative to the total pimonidazole-negative tumor area. The percentage of αB-crystallin in the hypoxic area was determined as the tumor area positive for αB-crystallin and pimonidazole relative to the total pimonidazole-positive area.

### αB-crystallin mRNA expression upon hypoxia

The HNSCC cell line UT-SCC-5 (described in [31]), maintained in DMEM + GlutaMAX (Invitrogen) supplemented with 10% fetal calf serum (Gibco-BRL) was seeded on 6-wells plates, 0.5×10^6^ cells per well, N = 4 per time point, and transferred after 24 hours from a standard humidified 37°C incubator to a humidified 37°C H35 hypoxystation (Don Whitley Scientific) with 0.1% O_2_. Samples were harvested after 0–22 hours of hypoxic incubation for quantitative RNA analysis.

### αB-Crystallin mRNA expression upon hypoxia, reoxygenation and N-acetylcysteine

The HNSCC cell lines UT-SCC-5 and UT-SCC-15 (described in [31]), maintained in DMEM + GlutaMAX supplemented with 10% fetal calf serum were seeded in 6-wells plates, 0.3×10^6^ cells per well, N = 4 per condition, and transferred after 24 hours from a standard humidified 37°C incubator to a H35 hypoxystation with 0.1% O_2_ (Don Whitley Scientific), or maintained in a standard incubator. Normoxic and hypoxic samples were harvested after 48 hours for quantitative RNA analysis. The reoxygenation samples were transferred after 24 hours of hypoxic incubation to a standard incubator again for 24 hours and subsequently harvested. To reduce ROS, cells were incubated after 24 hours of hypoxia or normoxia with 0.05 mM NAC (Sigma). After NAC was added, the reoxygenation samples were transferred from the hypoxystation to a standard humidified 37°C incubator and incubated for 24 hours and subsequently harvested.

### αB-crystallin protein expression upon hypoxia and reoxygenation

The UT-SCC-5 cell line was seeded in T25 flasks, 0.9×106 cells per flask, N = 3 for the normoxic condition and N = 4 for the hypoxic and reoxygenation condition, and incubated for 5 hours in a standard humidified 37°C incubator. The cells were transferred to the hypoxystation maintained at 0.1% or kept in the standard incubator and harvested after 48 hours. The reoxygenation samples were transferred after 24 hours of hypoxic incubation to the standard incubator again for 24 hours and subsequently harvested. The cells were harvested in 2% sodium dodecyl sulfate (Gibco), heated for 5 minutes at 100°C and sonicated 5× 30 seconds on and 30 seconds off with Bioruptor (Diagenode). The protein concentrations were determined with BCA Protein Assay Kit (Thermo Scientific) according to manufacturer’s protocol. Protein samples (60 μg/sample) were separated by electrophoresis on a 12.5% polyacrylamide gel and transferred to a nitrocellulose membrane (Protran). The membranes were blocked with 5% Elk (Campina) in PBS for an hour and washed 3 times for 10 minutes with PBS + 0.0025% v/v Nonidet P-40. The membranes were incubated for an hour with the 200-fold diluted monoclonal mouse-α-human-αB-crystallin (RIKEN) and 6000-fold diluted mouse-α-human-γ-tubulin as nce (Sigma-Aldrich) diluted in 0.025% w/v Nonidet P-40 and completed with 2% Elk in PBS. After washing, blots were incubated for 1 hour with a 6000-fold dilution of IRDye 800CW goat-α-mouse IgG (LI-COR). The proteins were visualized with the Odyssey scanner (LI-COR). Analysis was performed using Odyssey 2.1 software.

### αB-crystallin mRNA expression upon H_2_O_2_-induced oxidative stress

UT-SCC-5 cells were seeded in 6-wells plates, 0.5×10^6^ cells per well; N = 4 per concentration maintained in DMEM + GlutaMAX supplemented with 10% fetal calf serum. After 24 hours, cells were incubated with 0 mM (mock), 0.3 mM, 1.5 mM or 3.0 mM H_2_O_2_ for 1 hour after which they were incubated again in normal medium and harvested after 7 hours for quantitative RNA analysis.

### Hypoxia survival upon siRNA-mediated knock-down of αB-crystallin

4.4 ×10^6^ UT-SCC-5 cells were seeded in a T175 culture flask and maintained in DMEM + GlutaMAX supplemented with 10% fetal calf serum. After 24 hours, cells were transfected with siRNA using Lipofectamine 2000 Reagent according to manufacturers’ protocol (Invitrogen). The siRNA’swere directed against luciferase (siRNA LUC, sequence: CGUACGCGGAAUACUUCGAdTdT) and EGFP (si-EGFP, sequence: CGAGAAGCGCGAUCACAUGdTdT) as negative controls and αB-crystallin (si-αB1, sequence: GCACCCAGCUGGUUUGACAdTdT, si-αB2 sequence: CCCUGAGUCCCUUCUACCUdTdT and si-αB3, sequence: CCGGAUCCCAGCUGAUGUAdTdT). After 5 hours, cells were reseeded 4.0×10^4^ cells/well in 96-wells plates (6-fold per condition) and 1.25 ×10^5^ cells/well in 6-wells plates (4-fold per condition). The next day, the cells in the 96-wells plates were washed twice with PBS and DMEM (supplemented with GlutaMAX, 1 mM sodiumpyruvate and 10% fetal calf serum) was added containing 0 mM or 5 mM Glucose (Dextrose D(+), Invitrogen). After one hour, cells were kept in the standard incubator or transferred to the H35 hypoxystation maintained at 0.1% O_2_ and incubated for 24 hours. All 96-wells plates were subsequently incubated in the standard incubator for 3.5 hours and washed twice with PBS and incubated for two hours in 10-fold diluted Cell Counting Kit-8 solution (Sigma-Aldrich) in Optimem (Invitrogen). The absorbance at 450 nm was measured using an ELISA-reader (Tecan). The cells in the 6-wells plates were harvested 48 hours after siRNA transfection for quantitative RNA analysis to determine the efficiency of αB-crystallin mRNA knockdown.

### RNA analysis by quantitative RT-PCR

Total RNA from the harvested UT-SCC-5 and UT-SCC-15 cells was extracted using standard Trizol isolation. After DNAse I treatment (Amplification grade, Invitrogen) mRNAs were reverse transcribed using oligo (dT) primers and the Reverse Transcription System (Promega) according to manufacturer’s protocol starting with 1 μg of RNA in a final volume of 20 μl. Subsequently, quantitative PCR (qPCR) reactions were performed with 10 μl Power SYBR Green (Applied BioSystems), 5 μM primers and 2 μl cDNA in a final volume of 20 μl. The used primer sequences for αB-crystallin are: ATCTTCTTTTGCGTCGCCAG and TTCCCCATGGTGTCTGAGC, and for GAPDH: GATTGAGGTGCATGGAAAAC and AGGACCCCATCAGATGACAG. The fluorescent signal intensities were recorded with the ABI Prism 7000 system (Applied Biosystems). Samples were kept for 10 minutes at 95°C, followed by 40 cycles of 15 seconds at 95°C and 1 minute at 60°C. Data analysis was performed on the CFX96 (Biorad). Analysis was performed with CFX Manager Software (Biorad).

### Statistics

Statistical analyses were performed using Graphpad Prism 5.00 software. Statistical analysis was performed using One-way ANOVA and Tukey’s Multiple Comparison.

## Results

### αB-crystallin protein is increased in the hypoxic areas of HNSCC biopsies

**Figure 1:**
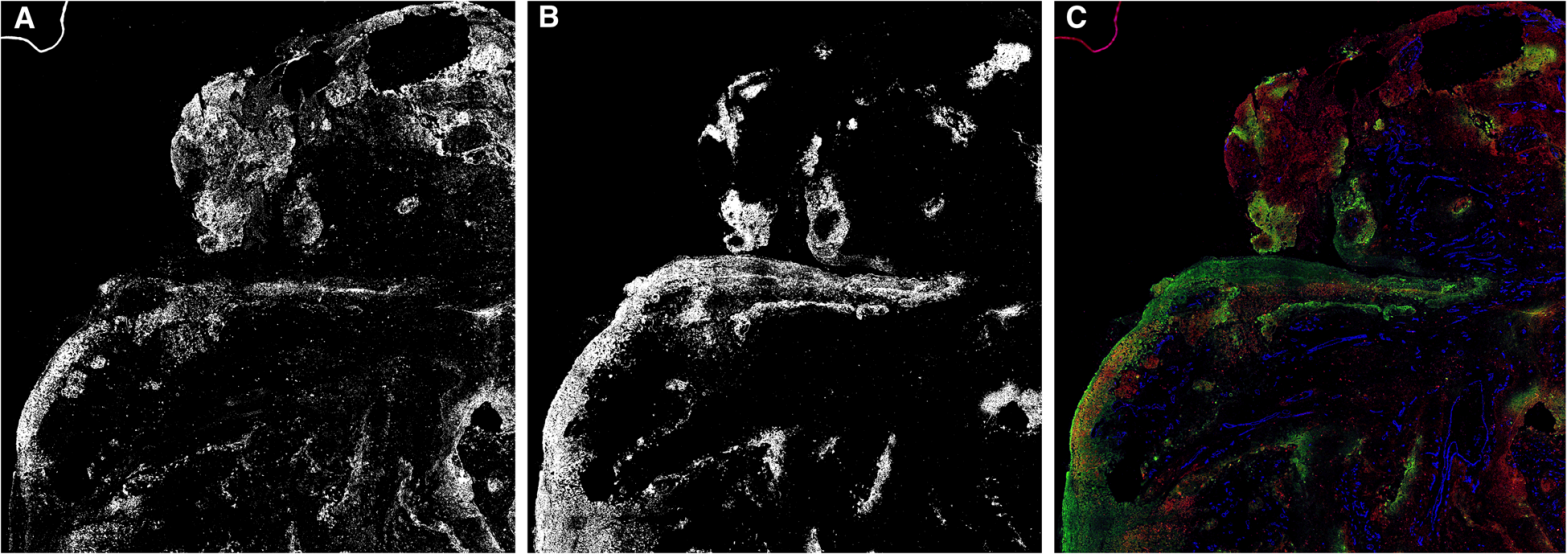
**Immunofluorescent staining of human HNSCC for αB-crystallin, pimonidazole-modified proteins and PAL-E.** Shown is a representative biopsy section. The fluorescent grey scale images were binarized, resulting in black and white images for αB-crystallin staining **(A)** and pimonidazole-modified proteins staining, indicating the hypoxic areas **(B)**. The merged image with αB-crystallin staining (assigned red), pimonidazole staining (assigned green) and PAL-E blood vessel staining (assigned blue) shows a substantial overlap between αB-crystallin and hypoxic regions. Hypoxic regions are mostly located in areas at greater distance from vessels **(C)**.

**Figure 2:**
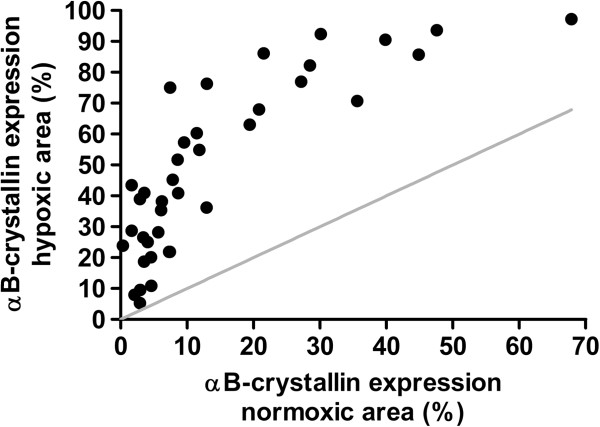
**αB-crystallin expression is increased in hypoxic areas.** The symbols represent the relative amount of αB-crystallin staining in the normoxic areas and in the hypoxic areas for each individual HNSCC. Equal staining of αB-crystallin in the normoxic and hypoxic areas would be according to the grey line.

The expression of αB-crystallin protein was analyzed in the hypoxic and normoxic regions present in sections of HNSCC biopsies of 38 different patients. To detect the hypoxic regions, the hypoxia marker pimonidazole was used. Figure 1 shows a representative binarized staining of αB-crystallin and pimonidazole (Figure 1A and 1B). Most of the hypoxic areas (Figure 1C, depicted in green) are located in tumor areas at larger distance from blood vessels (Figure 1C, depicted in blue). Interestingly, areas showing αB-crystallin expression (Figure 1C, depicted in red) are largely overlapping with hypoxic areas, though αB-crystallin can be detected in normoxic areas as well. By digital analysis of the scanned images the percentages of αB-crystallin-positive areas in the normoxic tumor areas and in the hypoxic tumor areas were determined for each biopsy. In case hypoxia does not affect αB-crystallin expression, the percentages of αB-crystallin-positive areas present in normoxic and hypoxic areas would be similar (Figure 2, grey line). However, as shown in Figure 2, the percentages of αB-crystallin-positive areas were found to be higher in the hypoxic than in the normoxic areas in all analyzed HNSCC biopsies.

### αB-crystallin expression is upregulated by reoxygenation, not hypoxia

**Figure 3:**
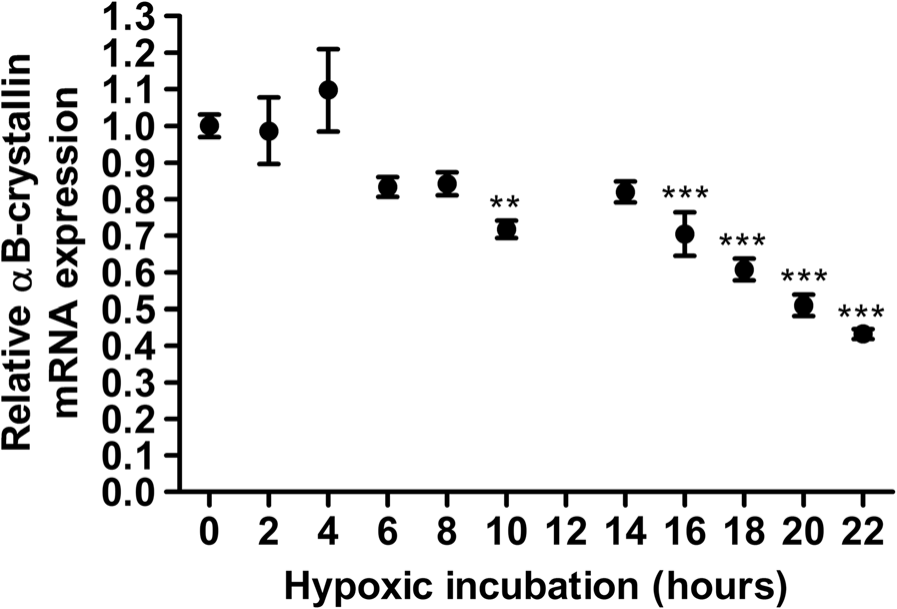
**Relative αB-crystallin mRNA expression during hypoxia.** αB-crystallin mRNA expression levels in UT-SCC-5 cells after incubation in a humidified 37CH35 hypoxystation at 0.1% O_2_ for the indicated time points. αB-crystallin mRNA expression levels were assessed via RT-qPCR (N = 4) *** p < 0.001, ** 0.001 < p < 0.01.

**Figure 4:**
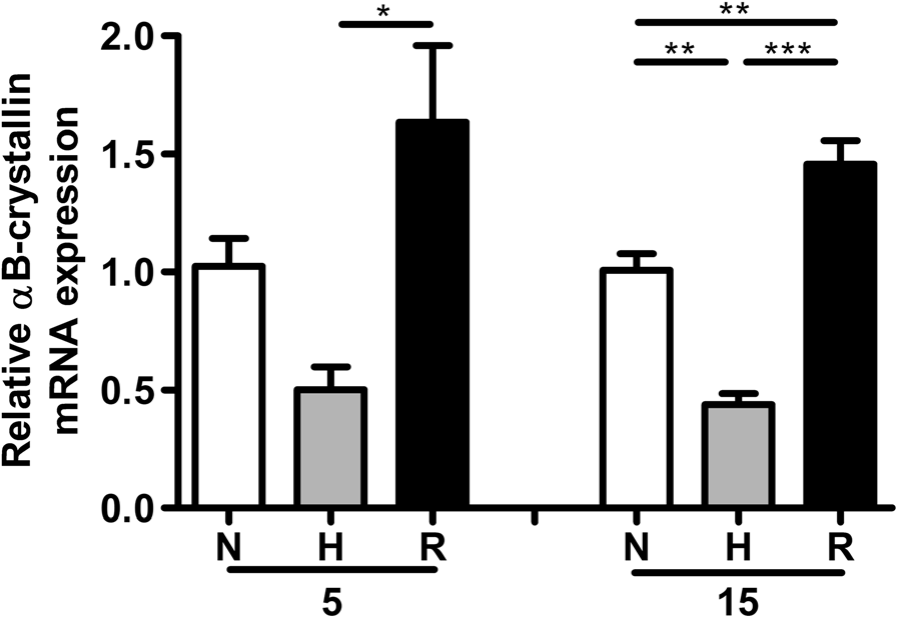
**Relative αB-crystallin mRNA levels after hypoxia and reoxygenation.** αB-crystallin mRNA levels in UT-SCC-5 and UT-SCC-15 cells under 48 hours normoxia (N), hypoxia (H, 0.1% O_2_) and after reoxygenation (R, 24 hours 0.1% O_2_/24 hours normoxia). αB-crystallin mRNA expression levels were assessed via RT-qPCR (N = 4). *** p < 0.001, ** 0.001 < p < 0.01, * 0.01 < p < 0.05.

The increased presence of αB-crystallin in the hypoxic areas might be the result of two different processes: a stress-induced upregulation of αB-crystallin and/or a longer survival of the cells expressing αB-crystallin. It has been shown that hypoxia stimulates upregulation of αB-crystallin in piglet stomach, colon and heart tissue [25,26]. For this reason we first tested whether hypoxic incubation is able to increase αB-crystallin mRNA expression levels of HNSCC cell lines by using quantitative RT-PCR. The HNSCC cell line UT-SCC-5 was maintained for 22 hours under 0.1% O_2_ conditions and every 2 hours (except t = 12 hours), αB-crystallin mRNA expression levels were determined. Surprisingly, αB-crystallin mRNA levels were found not to be upregulated, but actually 2.3-fold downregulated after 22 hours (Figure 3, P < 0.001), which suggests that the increased expression of αB-crystallin in the hypoxic areas of HNSCC is not directly caused by low oxygen levels. Reoxygenation can also lead to αB-crystallin upregulation, as has been shown in cultured optic nerve astrocytes [32]. Since in some areas hypoxia can be intermittent, resulting in periods with higher oxygen [2-4], we assessed the effect of reoxygenation on αB-crystallin expression. Consistent with the previous experiment, after 48 hours of hypoxic incubation, αB-crystallin mRNA expression was downregulated in UT-SCC-5 as well as in the HNSCC cell line UT-SCC-15 (Figure 4). However, upon reoxygenation, αB-crystallin expression levels were significantly higher than in the cells which were only incubated under normoxic conditions. A similar response was also observed in HeLa cells (data not shown), indicating that this might be a general response.

**Figure 5:**
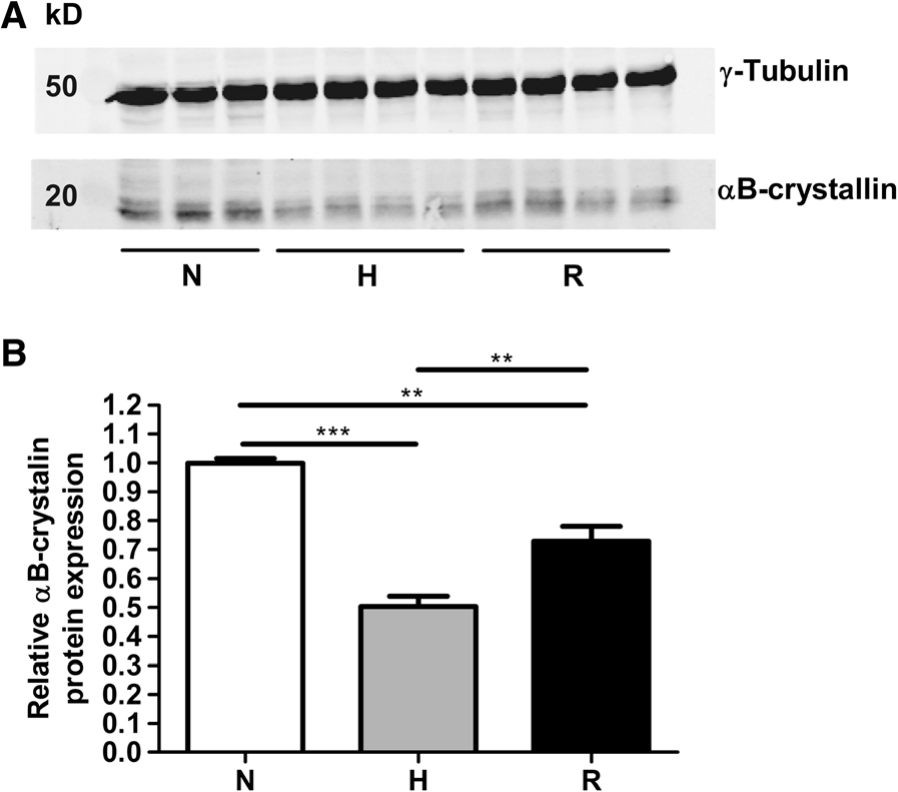
**Relative αB-crystallin protein levels after hypoxia and reoxygenation.** αB-crystallin protein expression levels in UT-SCC-5 cells under 48 hours normoxia (N), hypoxia (H, 0.1% O_2_) and after reoxygenation (R, 24 hours 0.1% O_2_/24 hours normoxia). αB-crystallin protein expression was analyzed of 3–4 independent incubations via western blotting **(A)** and quantified **(B)**. ***p < 0.001, ** 0.001 < p < 0.01.

Next we tested if a similar effect of the reoxygenation could be found on the protein level. For this we could only use the UT-SCC-5 cell line, since αB-crystallin expression in UT-SCC-15 cells was too low to allow detection by western blotting. After 48 hours of hypoxic conditions, αB-crystallin protein levels were decreased 2.0-fold (Figure 5, p < 0.001) and upon reoxygenation the αB-crystallin level was raised 1.5-fold compared to the hypoxic level (p < 0.01). Although the level after reoxygenation did not reach the level observed at normoxic conditions (p < 0.01), these results show that also the αB-crystallin protein was upregulated upon reoxygenation after hypoxia.

### αB-crystallin mRNA overexpression during reoxygenation is induced by ROS

**Figure 6:**
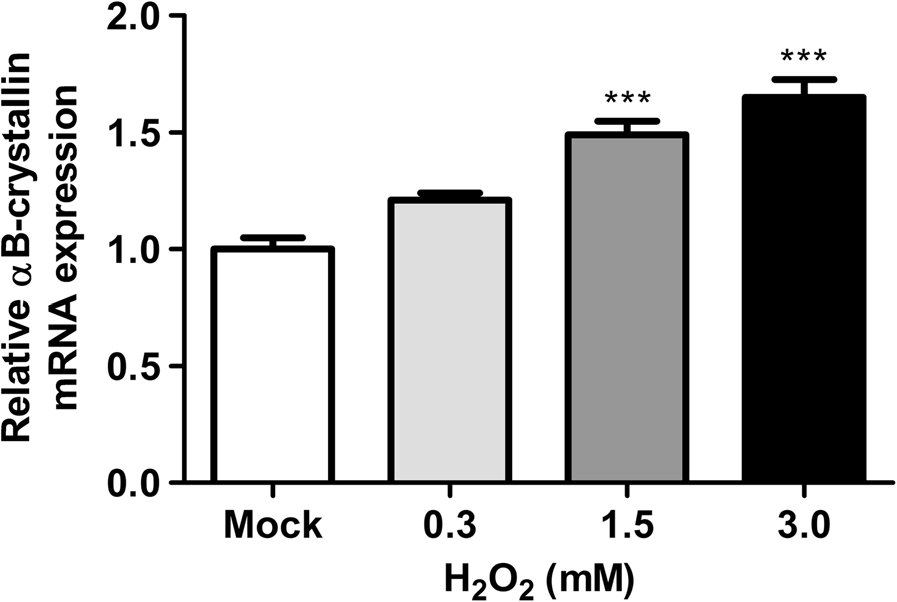
**Relative αB-crystallin mRNA levels upon H**_**2**_**O**_**2**_**-incubation.** Relative αB-crystallin mRNA levels in UT-SCC-5 cells after incubation with 0.0 mM (mock), 0.3 mM, 1.5 mM or 3.0 mM H_2_O_2_ for 1 hour and 7 hours of recovery. αB-crystallin mRNA expression levels were assessed via RT-qPCR (N = 4). *** p < 0.001.

**Figure 7:**
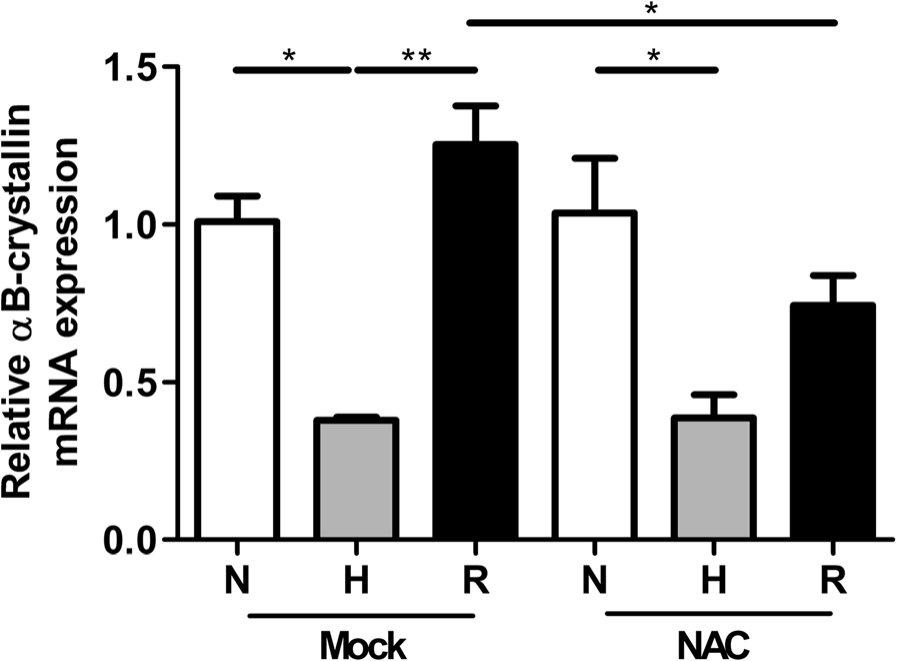
**Effect of the ROS-scavenger NAC on αB-crystallin mRNA levels during reoxygenation.** UT-SCC-5 cells after incubation with mock or NAC under 48 hours normoxia (N), hypoxia (H, 0.1% O_2_) and after reoxygenation (R, 24 hours 0.1% O_2_/24 hours normoxia). ** 0.001 < p < 0.01, * 0.01 < p < 0.05.

Reoxygenation stimulates the production of ROS [33], which at a high concentration is stressful for cells. As a reaction, cells can protect themselves by increasing the level of stress proteins [32]. To test whether ROS induces αB-crystallin expression in HNSCC cells, UT-SCC-5 cells were treated with H_2_O_2_. The cells were incubated for 1 hour with a H_2_O_2_ concentration series between 0 and 3.0 mM H_2_O_2_ and subsequently harvested after 7 hours (Figure 6). At the protein level no effect could be detected, because the protein expression was too low to allow accurate measurement by western blotting (data not shown). However, a significant increase in αB-crystallin mRNA expression could be observed at 1.5 mM and 3.0 mM H_2_O_2_, compared to incubation with mock (P < 0.001). Next, it was tested whether induction of αB-crystallin upon reoxygenation can be reduced with the ROS scavenger NAC. UT-SCC-5 cells were incubated without and with NAC during normoxia, hypoxia and reoxygenation. Without NAC, αB-crystallin mRNA levels were again downregulated during hypoxia and upregulated during reoxygenation, as expected (Figure 7). In the presence of NAC, the hypoxia-induced αB-crystallin mRNA downregulation remained the same, whilst the upregulation upon reoxygenation was reduced 1.7-fold, compared to reoxygenation without NAC. These results suggest that the ROS produced upon reoxygenation is at least in part responsible for the upregulation of αB-crystallin in UT-SCC-5 cells.

### αB-crystallin knockdown leads to diminished cell survival under hypoxic and hypoglycemic stress

**Figure 8:**
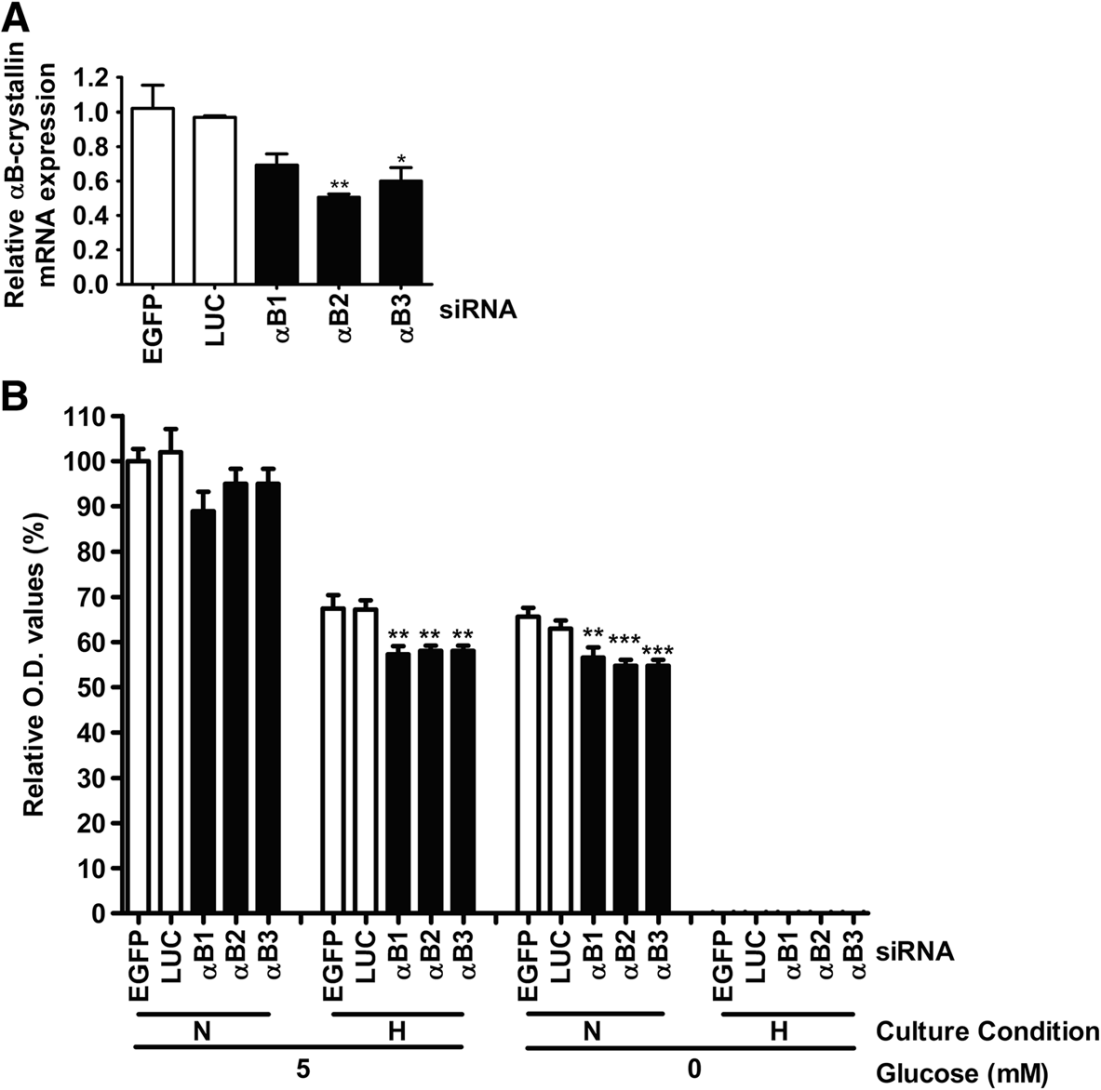
**Knockdown of αB-crystallin expression reduces hypoxia and hypoglycemia survival.** Expression of αB-crystallin mRNA in UT-SCC-5 cells was reduced by three different αB-crystallin siRNAs (αB1, αB2 and αB3). LUC and EGFP were used as negative control siRNAs **(A)**. Survival of siRNA-treated UT-SCC-5 cells under normoxic (N) and hypoxic (H, 0.1% O_2_ for 24 hours) conditions in the presence of 5 mM or 0 mM glucose **(B)**. Cell survival was assessed via a colorimetric assay using cell counting kit-8. The optical density (O.D.) of siEGFP-treated cells was set at 100%. *** p < 0.001, ** 0.001 < p < 0.01, * 0.01 < p < 0.05.

Next, it was investigated whether cells expressing αB-crystallin were able to survive longer under hypoxic conditions. In hypoxic areas, not only a shortage in oxygen, but hypoglycemia as well is a physiological stressor [34]. Since glucose is able to protect cells under hypoxic conditions [35] and glucose is the main energy source required for HNSCC cell survival [36,37], medium without glucose was used as an additional stress condition. Reduction of αB-crystallin expression in UT-SCC-5 cells was obtained by siRNA-mediated knockdown. Cells with normal and reduced levels of αB-crystallin were exposed to a hypoxic oxygen level of 0.1% for 24 hours, after which the survival was determined by analyzing the cell number with Cell Counting Kit-8. The knockdown of αB-crystallin was performed with 3 different siRNAs and compared with 2 different control siRNAs (Figure 8A). Under normoxic conditions in the presence of 5 mM glucose, knockdown of αB-crystallin did not significantly alter cell survival, although with all three siRNAs a trend in survival reduction was observed (Figure 8B). Hypoxic stress in the presence of 5 mM glucose, led to a significant lower cell survival, compared to normoxic conditions (67% for siEGFP and siLUC). Cell survival could be further reduced significantly, by knockdown of αB-crystallin (57% for si-αB1 and 58% for si-αB2 and si-αB3). Under normoxic conditions, 0 mM glucose led to lower cell survival rates (66% for siEGFP and 63% for si-LUC), which was further reduced as well after knockdown of αB-crystallin (57% for si-αB1 and 55% for si-αB2 and si-αB3). Combining hypoxic as well as hypoglycemia stress was detrimental resulting in 0% cell survival. Since all 3 different αB-crystallin siRNAs gave similar results, it is very unlikely that these observations are due to off-targets effects. These results show that reduction of the αB-crystallin level decreases the survival of hypoxia-exposed and glucose-deprived cells.

## Discussion

In the current study, we show that more αB-crystallin protein is present in hypoxic HNSCC tumor areas than in normoxic areas. Since an increased αB-crystallin expression might be the result of a stress-induced transcriptional upregulation, we investigated whether hypoxic stress is able to induce αB-crystallin expression in HNSCC cell lines. Remarkably, under hypoxic conditions αB-crystallin mRNA expression was found to be downregulated in UT-SCC-5 and UT-SCC-15 cells. It is not clear how αB-crystallin is downregulated, but this could be due to a general transcription shutdown caused by epigenetic modifications [38,39]. Only after reoxygenation, a significant upregulation of αB-crystallin mRNA in the HNSCC cell lines was found. The same trend was observed at the protein level. The upregulation of αB-crystallin has also been observed in other cell types and by different forms of reoxygenation stress, such as chemical ischemic/recovery stress and ischemic/reperfusion injury [40,41], although from those studies it appeared that the upregulation of αB-crystallin is not a general mechanism since not all cell types show this effect [40]. The reoxygenation-induced upregulation of αB-crystallin also fits with the studies performed with the piglets, where hypoxia-induced αB-crystallin upregulation was detected [25,26]. In these studies piglets were maintained in a hypoxic chamber for either 1 or 4 hours and were allowed to recover over periods of 1 to 68 hours under normoxic condition and thus underwent a period of reoxygenation.

The reoxygenation-induced upregulation of αB-crystallin mRNA is at least partially caused by ROS, based on the inhibitory effect of the ROS-scavenger NAC (Figure 7). Despite the low level of oxygen, significant levels of ROS can be present in hypoxic areas and thus can be responsible for the induction of αB-crystallin expression. ROS-levels in hypoxic areas can be increased by moments of reoxygenation due to intermittent, perfusion-limited hypoxia [9] or produced by necrotic cells which are often present in hypoxic tumor areas [34,42-44]. Also ROS can be produced by a synergistic effect of oncogenic-induced stimulation of increased mitochondrial capacity and low oxygen levels, which causes an ineffective functioning of mitochondrial respiratory complexes [10]. For this a hypoxia-induced downregulation of thioredoxin reductase 1 seems to be important in maintaining high levels of ROS under hypoxic conditions [45]. As mentioned earlier, some normoxic areas may also contain significant levels of αB-crystallin. These local αB-crystallin expressions might be explained by intermittent hypoxia as well. As shown by Bennewith and colleagues a substantial proportion of tumor cells can go through cycles of hypoxia and normoxia [46,47]. If the intervals of hypoxia are too short or if pimonidazole is not present at the hypoxic moments, erroneously no staining by this marker will be detected [46,47]. Nevertheless, αB-crystallin induced by the ROS formed during the reoxygenation periods might still be present.

αB-crystallin is a stress protein which may enhance cell survival upon ROS exposure, as shown for H_2_O_2_ treated mouse retinal pigment epithelium cells [48]. Based on knockdown experiments, we showed that αB-crystallin is able to play a role in the survival of cells coping with hypoxia and glucose-deprivation stress as well. It is thus possible that the αB-crystallin present in hypoxic tumor areas plays a role in tumor cell survival during hypoxic stress [34,49]. This protective activity of αB-crystallin may further increase the number of αB-crystallin-positive cells in hypoxic tumor areas. In summary, the relative higher levels of αB-crystallin in HNSCC hypoxic tumor areas might be caused by a combination of ROS-induced αB-crystallin upregulation and an enhanced survival of αB-crystallin-positive cells exposed to this stress.

The enhanced expression of αB-crystallin in HNSCC may have a negative effect on the prognosis of the patient. We have recently found that αB-crystallin expression is associated with distant metastases formation in HNSCC patients [19]. This association might relate to the chaperone function of αB-crystallin in mediating folding and secretion of VEGF. αB-crystallin is able to bind misfolded vascular endothelial growth factor (VEGF), leading to enhanced VEGF secretion [50,51]. VEGF is specifically upregulated by hypoxia-inducible factor 1 (HIF1) and is important for tumor vascularization. VEGF induction is thus a mechanism to alleviate hypoxic circumstances [52,53]. Cycling hypoxia-induced VEGF expression has been shown to increase pulmonary metastasis formation in mice [54]. Since αB-crystallin can increase hypoxic cell survival and can help in the (re)folding of hypoxia-induced VEGF expression, αB-crystallin expression could ultimately increase the risk of hypoxic tumors to become metastasis-prone [55]. Furthermore, by increasing hypoxic cell survival αB-crystallin may also decrease the sensitivity of a tumor to cancer treatments, such as radiation or other cancer treatments, as shown by the effect of αB-crystallin on tumor necrosis factor-related apoptosis-inducing ligand (TRAIL) as well as cisplatin-induced apoptosis in human ovarian cancer cells [56]. Because of its potential to interfere with anti-tumor therapies, αB-crystallin might be a promising target for anti-cancer treatment.

## Conclusions

Enhanced αB-crystallin expression in HNSCC and also in other kind of tumors correlates with poor prognosis of the patients. The underlying stress that induces αB-crystallin expression in HNSCC was not known. Here we show that αB-crystallin is most abundantly present in the hypoxic areas of the tumor, likely caused by ROS stress. The increased expression of αB-crystallin may lead to prolonged survival of hypoxic cells, thereby protecting those cells which are most resistant against cancer treatments.

## Abbreviations

HIF1: Hypoxia-inducible factor 1; HNSCC: Head and neck squamous cell carcinoma; NAC: N-acetylcysteine; ROS: Reactive oxygen species; TRAIL: Tumor necrosis factor-related apoptosis-inducing ligand; VEGF: Vascular endothelial growth factor.

## Competing interests

The authors have no conflicts of interest to declare.

## Authors’ contributions

CS participated in the study concept and design, data acquisition of all figures, data analysis and interpretation, statistical analysis, manuscript preparation and editing. ES participated in the data acquisition of Figures 3, 4, 5 and 6, data analysis and interpretation and statistical analysis. JB participated in the study concept and design and manuscript editing. PS participated in study design, data analysis and interpretation, statistical analysis and manuscript editing. RG participated in data acquisition of Figures 3, 4, 5, 6 and 7 and in manuscript reviewing. GP participated in study concept and manuscript reviewing. JK participated in study concept and design and manuscript editing. WB participated in study concept and design, data analysis and interpretation and manuscript editing. All authors read and approved the final manuscript.

## Pre-publication history

The pre-publication history for this paper can be accessed here:

http://www.biomedcentral.com/1471-2407/14/252/prepub
